# Interband plasmonic nanoresonators for enhanced thermoelectric photodetection

**DOI:** 10.1515/nanoph-2024-0752

**Published:** 2025-03-28

**Authors:** Golnoush Zamiri, Simon Wredh, Md Abdur Rahman, Nur Qalishah Adanan, Cam Nhung Vu, Hongtao Wang, Deepshikha Arora, Haruya Sugiyama, Wakana Kubo, Zhaogang Dong, Robert E. Simpson, Joel K.W. Yang

**Affiliations:** Singapore University of Technology and Design, 8 Somapah Road, Singapore 487372, Singapore; Tokyo University of Agriculture and Technology, Faculty of Engineering, Koganei, Tokyo, Japan; Institute of Materials Research and Engineering (IMRE), Agency for Science, Technology and Research (A*STAR), 2 Fusionopolis Way, Innovis #08-03, Singapore 138634, Republic of Singapore; Quantum Innovation Centre (Q.InC), Agency for Science Technology and Research (A*STAR), 2 Fusionopolis Way, Innovis #08-03, Singapore 138634, Republic of Singapore; School of Engineering, University of Birmingham, Edgbaston, Birmingham, B15 2TT, UK; Singapore-HUJ Alliance for Research and Enterprise (SHARE), The Smart Grippers for Soft Robotics (SGSR) Programme, Campus for Research Excellence and Technological Enterprise (CREATE), Singapore 138602, Singapore

**Keywords:** plasmonic nanoresonators, thermoelectric photodetector, interband plasmonics, photo-thermoelectric effect, cascaded device

## Abstract

Thermoelectric photodetectors are robust alternatives to photodiodes with applications in extreme environments; however, the poor absorptivity of thermoelectric materials limits their photosensitivity. Here, we take a new look at the traditional thermoelectric materials Sb_2_Te_3_ and Bi_2_Te_3_ in their recently discovered ability to support interband plasmonic resonances in the visible spectrum. We fabricated nanoresonators directly into the thermoelectric materials to improve their optical absorptance through plasmonic field enhancements, leading to improved photo-thermoelectric conversion. A thermoelectric detector with Sb_2_Te_3_ and Bi_2_Te_3_ nanostructures demonstrated ∼90 % optical absorptance across the visible spectrum, more than twice that of unpatterned materials. The solid-state device was fabricated on a substrate and exhibited a response time of 160 µs and a specific detectivity of $3.2\times 10^{6}\;\text{cm H}{\text{z}}^{1/2}\;{\text{W}}^{-1}$ . Our demonstration that plasmonic and thermoelectric properties can be exploited within the same material could advance photodetectors and other optoelectronic technologies, such as biosensors, solar cells, and integrated spectrometers.

## 1 Introduction

Photodetection – the conversion of photons into an electrical signal for the purpose of detecting electromagnetic radiation – has a wide range of applications including imaging, optical communication, optical instrumentation, and solar cells. In the visible spectrum, photodiodes based on photoexcitation in semiconductors are commonly used for their high speed and sensitivity [1]. An alternative to photodiodes is thermoelectric detectors with certain advantages, such as broadband sensitivity from ultraviolet to far-infrared, greater resilience to electromagnetic interference and temperature fluctuations, and the ability to operate without external power [2], [3], [4], [5], [6]. The reliability of thermoelectric devices has made them the superior choice for extreme environments and they are, for instance, found aboard space vehicles. These devices exploit the thermoelectric effect, where a photo-induced temperature difference in a material generates an electric potential difference [6]. On the downside, the electrical signal generated by the thermoelectric effect is relatively weak compared to photoexcitation in semiconductors, and thermoelectric detectors are thus limited by their low detectivity [2].

Detectivity can be enhanced by utilizing high *zT* thermoelectric materials, miniaturizing the device, and increasing optical absorptance. Telluride compounds, such as Sb_2_Te_3_ and Bi_2_Te_3_, are well known for their outstanding thermoelectric properties. These materials exhibit high Seebeck coefficients and low thermal conductivity, which are essential for efficient thermoelectric conversion [7]. These properties make them highly effective in generating electrical power from temperature gradients and in applications requiring heat-to-electricity conversion. Because of these advantages, Sb_2_Te_3_ and Bi_2_Te_3_ have been used in thermopile photodetectors [5], [8], [9], [10]. However, the photo-thermoelectric performance of these materials is limited by poor optical absorptance of ∼40 % in the visible range, which is essential for generating a strong electrical photoresponse. Enhancing the optical absorptance in thermoelectric devices is thus a key factor that can improve detection efficiency.

Previous efforts to increase optical absorptance have employed indirect approaches, where a highly absorptive blackened material or structure is coated onto the thermoelectric materials. For instance, metallic metasurface absorbers have been added onto thermoelectric materials to enhance detectivity [2], [8], [11]. As these absorbers heat up from light absorption, thermal conduction into adjacent or underlying thermoelectric materials then increases its temperature. A consequence of indirect heating may be a reduced responsivity of the photodetectors. Adding an absorbing material increases the total heat capacity of the device, leading to reduced temperatures and slow response times. Nonetheless, these results indicate that plasmonic properties of noble metals can be exploited to concentrate and trap light within the active material, effectively increasing the absorption cross section and improving device performance [12]. Additionally, the tunability of plasmonic nanoresonators allows for precise control over the absorption spectrum, enabling optimization for specific applications and wavelengths of interest.

Intriguingly, p-block compounds, such as Sb_2_Te_3_ and Bi_2_Te_3_ and even p-block elements like Si, have recently received attention because features in their electronic band structure lead to strong interband transitions and plasmonic-like properties [13], [14]. This combination of plasmonic-like and thermoelectric properties in the same materials is relatively unexplored [15] yet it provides an opportunity for novel device designs with enhanced functionality. By fabricating plasmonic nanoresonators directly out of the thermoelectric materials, localized heating and large thermal gradients can be achieved by optical excitation. Due to the Seebeck effect, these thermal gradients lead to a nonuniform distribution of charge carriers and a measurable potential difference. A nanoresonant thermoelectric detector was previously demonstrated with a narrowband absorptance of ∼60 % directly in Bi_2_Te_3_ and Sb_2_Te_3_ nanowires [5]. To improve the performance of thermoelectric detectors, it is desirable to further enhance the optical absorptance across the entire visible spectrum and beyond, e.g., for applications in photoelectric energy conversion.

In this work, we exploit the confluence of plasmonic-like and thermoelectric properties in the same materials (Sb_2_Te_3_/Bi_2_Te_3_) to design a responsive thermopile detector. By fabricating plasmonic nanoresonators directly out of thermoelectric materials, we enhanced optical absorptance to ∼90 % across the visible spectrum and achieved direct photo-thermoelectric conversion. Furthermore, nanoscale plasmonic effects could enable miniaturized thermopile devices with greatly enhanced responsivities. A cascaded thermopile detector consisting of five thermocouple junctions, featuring nanoresonators composed of Sb_2_Te_3_/Bi_2_Te_3_, exhibits a responsivity of 1.5 V/W for illumination at 650 nm.

## 2 Methods

### 2.1 Nanostructure fabrication and characterization

Silicon-oxide (SiO_2_) glass substrates were cleaned using acetone, isopropanol, and deionized water. Oxygen plasma treatment was performed to ensure surface activation and to remove any residual organic contaminants. We spin coated hydrogen silsesquioxane (HSQ) 6 % XR1541 at 3,000 rpm for 60 s to achieve 100  nm thick films on the glass substrate. A conductive layer consisting of 10  nm aluminum was deposited on top of the HSQ resist using electron-beam evaporation. Subsequently, nanoposts were patterned on the coated substrate via electron-beam lithography (Raith eLine) with an accelerating voltage of 30  kV and a beam current of 405 pA. The dose used for the nanopost structures was 2,500 μC cm^−2^. After exposure, a salty developer solution was employed to develop the structures, effectively removing the Al layer [16]. To remove the 10  nm Al layer, the substrate was immersed in the salty developer for 10  s. After this initial immersion, the sample was kept in the developer for an additional 2  min. Scanning electron microscope (SEM) imaging (JEOL JSM-7600F system) was used to examine the structural characteristics of the nanostructures. Absorptance measurements were conducted with a CRAIC microspectrophotometer, using unpolarized light and a 0.45 NA objective lens.

### 2.2 Device fabrication

**Figure 1:**
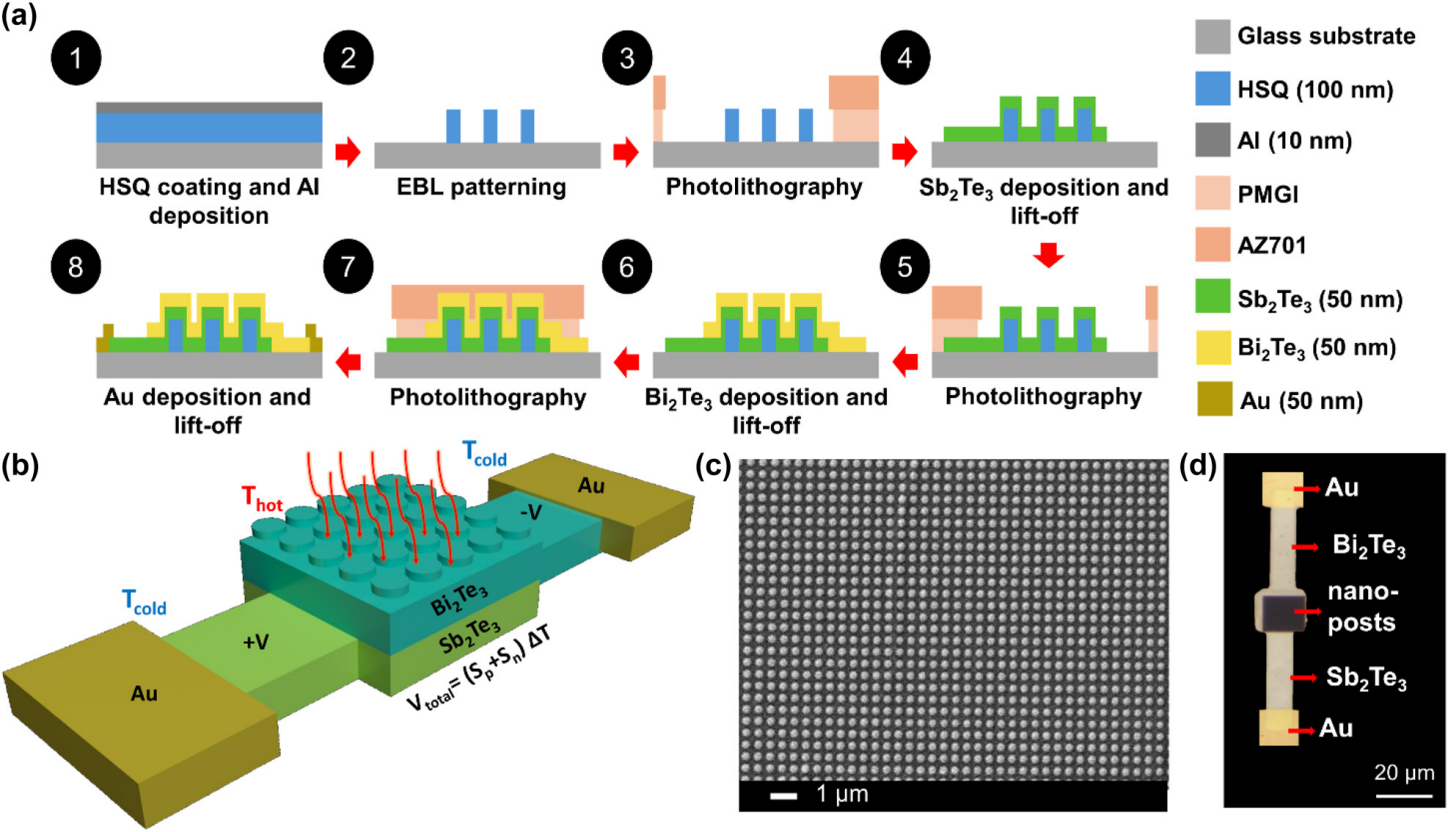
Fabrication process, schematic, and fabricated Sb_2_Te_3_/Bi_2_Te_3_ photodetector. (a) Fabrication process flow. The HSQ nanoposts are patterned using electron beam lithography. The remaining parts of the device are fabricated using maskless photolithography, magnetron sputtering (RF for tellurides and DC for Au), and liftoff. (b) Schematic of the thermoelectric detector showing temperature and voltages generated from light illumination. A nanostructure absorber array is incorporated at a Sb_2_Te_3_/Bi_2_Te_3_ thermocouple junction forming the active area of the detector to enhance its optical absorptance. Strong confinement of visible light directly in the thermoelectric materials provides an efficient conversion of photon energy to heat. A temperature difference builds up in the device and produces a Seebeck voltage difference across the thermocouple. (c) Scanning electron micrograph of fabricated Sb_2_Te_3_/Bi_2_Te_3_ nanostructures. (d) Micrograph of a fabricated single-unit device. The dark squares are the nanostructure absorbers, from which Sb_2_Te_3_ and Bi_2_Te_3_ legs are extended outward. The gold contact pads are used for electrical measurements.

Figure 1(a) illustrates the process of device fabrication. The devices were fabricated through three photolithographic cycles, with a lift-off process performed after the deposition of each layer. To ensure a clean liftoff, a bilayer of positive photoresists (PGMI and MIR 701) was spin coated on the fabricated nanostructures. The device structures were patterned using a Nanyte BEAM direct laser writing system. The patterned structures were developed in AZ726MIF for 60 s. And 50 nm thin films of Sb_2_Te_3_ and Bi_2_Te_3_ were deposited through different aligned photolithography and liftoff steps by RF magnetron sputtering using an AJA Orion 5 system. The deposition power and pressure were set to 20  W and 0.5  Pa, respectively. To crystallize the thin film of Sb_2_Te_3_, the sample was annealed at 150  °C for 5  min. For electrode connections, we utilized the same sputtering system to deposit a 50  nm thick gold (Au) film using the DC gun with a power of 50  W and deposition pressure of 0.5  Pa. The deposition of the films was done in a base vacuum pressure of 2.7 × 10^−5^  Pa. The final step involved a 1 h liftoff process in N-methylpyrrolidone (NMP) at 60  °C. After device fabrication, the substrate was wire bonded onto a printed circuit board (PCB), and the device electrodes were wire bonded to the PCB pads.

### 2.3 Electromagnetic simulations

Near-field and far-field electromagnetic simulations were performed in Lumerical using the finite-difference time-domain technique. A nanostructure unit cell was defined based on the geometry of SEM images and using measured optical constants. Periodic boundary conditions were applied to extend the structure into a periodic lattice, and a plane wave source was used to excite the structures. The size of mesh cells around the area of the nanostructure was constrained to 2 nm.

### 2.4 Thermoelectric simulations

Thermoelectric simulations were performed using the Heat Transfer and ACDC modules in Comsol Multiphysics. The device was placed on a thick glass substrate and encapsulated in an air box with constant temperature boundary conditions. No direct optical excitation was included in the simulations. Rather, heat sources were applied to the patterned and unpatterned regions of the devices with powers corresponding to the experimental light intensities and absorptance data. The time-dependent simulations were run for 10  ms such that the temperatures and thermoelectric potentials had time to reach steady state.

### 2.5 Photoresponse measurements

Photo-induced voltage measurements were carried out at room temperature, using a Keithley 2612B source meter. For focused monochromatic illumination, 405 nm and 650 nm diode lasers with Gaussian beam profiles and output powers of 1.0  mW were focused onto the absorber area using a 10x 0.25 NA lens. For uniform monochromatic illumination, a collimated 650  nm beam was used with an output power of 1.4–19.7  mW and a spot diameter of 2.4  mm. For uniform white-light illumination, a Nikon Halogen 12V50W LV-LH50PC lamp was used with a power of 2.8  mW and spot diameter of 0.42 mm. The incident light intensities *I*_0_ were calculated using $I_{0}=\frac{2P_{0}}{\pi r^{2}}$ for the Gaussian laser beam and $I_{0}=\frac{P_{0}}{\pi r^{2}}$ for the white-light illumination, where *P*_0_ is the power and *r* is the spot radius. See Supplementary Information for more details.

## 3 Results and discussion

To exploit the plasmonic properties of the material for enhanced optical absorption, nanoresonators were fabricated by depositing thermoelectric materials on top of dielectric nanoposts. Figure 1(a) and (b) illustrates a schematic of the fabrication process flow and the final device. We fabricated plasmonic nanoresonators composed of Sb_2_Te_3_ and Bi_2_Te_3_ by sputter deposition on an array of hydrogen silsesquioxane (HSQ) nanoposts that were defined using electron beam lithography, forming the active area of the thermopile photodetector. The plasmonic nanoresonators are incorporated into a thermopile structure, which consists of two electrodes: a thermoelectric *p*-type leg made from Sb_2_Te_3_, and a thermoelectric *n*-type leg made from Bi_2_Te_3_.

When the photodetector is exposed to visible light, the nanoresonators enhance the absorption of incident photons due to the plasmonic properties of Sb_2_Te_3_ and Bi_2_Te_3_. Photothermal conversion leads to localized heating at the active area of the detector. The localized heating creates a temperature difference between the nanoresonator area and the cool (room temperature) ends of the legs. In the *p*-type Sb_2_Te_3_ leg, holes diffuse toward the cooler end, while in the *n*-type Bi_2_Te_3_ leg, electrons diffuse toward the cooler end, resulting in the creation of a voltage difference *V* = (*S*_*p*_ + *S*_*n*_) Δ*T*, where Δ*T* is temperature difference, and *S*_*p*_ and *S*_*n*_ are Seebeck coefficients of the *p* and *n* type thermoelectric materials [5], [11]. The voltage difference is measured through the deposited gold electrodes and contact pads. This voltage signal corresponds to the intensity of the absorbed light, enabling photodetection based on the generated electrical signal.

To investigate the optical properties of thermoelectric nanoresonators, 50 nm of Sb_2_Te_3_ and 50 nm of Bi_2_Te_3_ were deposited on the HSQ nanoposts. Figure 1(c) shows the SEM image of nanoposts with a 300 nm pitch after deposition of Sb_2_Te_3_ and Bi_2_Te_3_, increasing the width of the nanoposts from 60 nm to approximately 160 nm wide.

**Figure 2:**
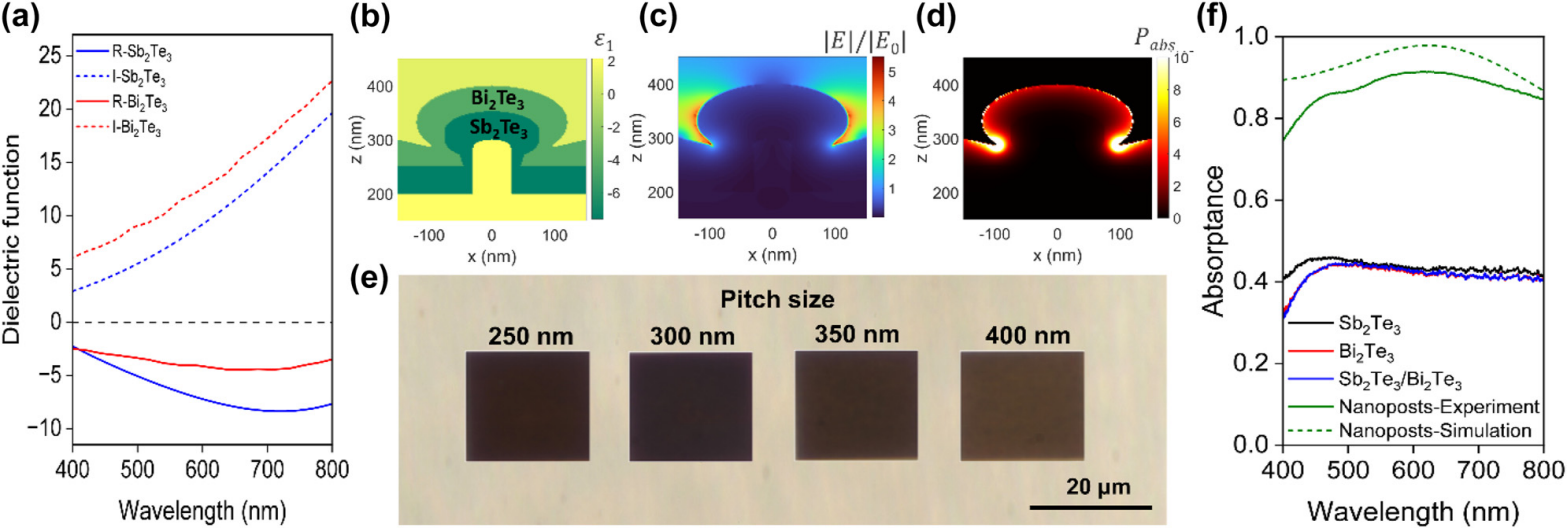
Optical properties of Sb_2_Te_3_/Bi_2_Te_3_ nanostructures. (a) Measured dielectric function of Sb_2_Te_3_ and Bi_2_Te_3_. The negative real permittivity indicates plasmonic behavior for visible wavelengths. (b–d) Real permittivity, electric field intensity normalized to the incident plane wave, and absorbed power density per unit volume (a.u.) of Sb_2_Te_3_/Bi_2_Te_3_ nanostructures at *λ* = 625 nm. (e) Optical micrographs of fabricated nanostructure arrays with varying pitch and fixed pillar diameters of 60 nm. The lower pitch structures are darker because of their larger absorptance in the visible spectrum. (f) Experimental and simulated absorptance spectra for Sb_2_Te_3_/Bi_2_Te_3_ nanostructure arrays with HSQ nanopost diameters of 60 nm and a pitch size of 300 nm.

Figure 2(a) shows the dielectric functions of Sb_2_Te_3_ and Bi_2_Te_3_. Both materials are expected to exhibit plasmonic-like behavior [17], which arises for wavelengths where the real part of the permittivity, *ε*′(*ω*), is negative. Despite not exhibiting as large a negative permittivity as other plasmonic materials such as Ag or Au, the permittivities of Sb_2_Te_3_ and Bi_2_Te_3_ do become negative in the visible light range for wavelengths between 400 and 800 nm. Therefore, by designing Sb_2_Te_3_ and Bi_2_Te_3_ nanostructures in which highly lossy plasmonic modes can be optically excited, photo-thermoelectric conversion can be enhanced for detection of visible light.

To gain insights into the near field-interactions between incident light and the nanoresonators, full-wave electromagnetic simulations using finite-difference time-domain (FDTD) were conducted. Periodic unit cells of the bilayer thermoelectric structures were modeled based on the nanostructure geometry from SEM inspection and the measured dielectric functions. The cross-sectional simulation model of the nanoposts made of Sb_2_Te_3_/Bi_2_Te_3_ is shown in Figure 2(b), where the color bar corresponds to the real permittivity at 625 nm wavelength. The dark green and light green regions represent 50 nm Sb_2_Te_3_ and Bi_2_Te_3_, respectively. Due to the nondirectional nature of sputter deposition, the sidewalls of the nanoposts are also coated, resulting in a mushroom-shaped structure [18]. The E-field intensity of the Sb_2_Te_3_/Bi_2_Te_3_ nanoposts is shown in Figure 2(c), and notably, the field is strongly localized around the perimeters of the structures, similar to the behavior of gold or silver ellipsoid nanoparticles [13]. Field localization and enhancement arising from plasmon resonances result in light absorption at specific hot spots on the structure. The observation of strong electric field localization at the edges of the nanostructures suggests a similar behavior in plasmonic semiconductors [19].

Figure 2(d) shows the calculated absorbed power density within the dual-layer structures. Adding a layer of Bi_2_Te_3_ significantly alters the absorbed power density distribution. The dual-layer structure exhibits enhanced absorption, with a more uniform distribution of absorbed power compared to the single-layer nanoposts (Figure S1(a)–(c)). This change suggests that the combination of materials in a layered structure can improve light absorption efficiency, potentially leading to better performance in photodetection applications. Notably, the absorbed power density is highly localized within the nanostructure, with the highest absorption occurring at the base of the structure. While the absorption is localized, it is reasonable to assume the temperature distribution becomes homogeneous within the nanostructures in tens of nanoseconds (see Supplementary Information), due to the small thermal diffusion length scale [20].

The effects on the optical response for different HSQ nanopost diameters, pitch size, and annealing conditions for single-layer Sb_2_Te_3_ structures were investigated prior to device fabrication, as shown in Figure S2(a)–(c). The results demonstrate that nanoposts with a diameter of 60  nm and a 300  nm pitch exhibited the strongest light absorption, and these parameters were, therefore, chosen for the dual-layer devices. Additionally, nanoring structures were fabricated, as shown in Figure S3(a)–(c). Similar optical measurements were conducted on these nanorings, revealing lower absorptance compared to the nanopost structures.

To further investigate the effect of dual-layer structures on optical absorptance, 50  nm of Sb_2_Te_3_ and Bi_2_Te_3_ were deposited on the nanostructures with a 60  nm diameter and 300  nm pitch. Optical absorptance spectra of the fabricated nanoresonators together with nonpatterned reference spectra of the thermoelectric films are shown in Figure 2(f). The results demonstrate a significant enhancement in optical absorptance when Sb_2_Te_3_ and Bi_2_Te_3_ are fabricated as nanostructures compared to as plain films. Both the simulated and experimental spectra of the nanoresonators show a broad resonance peak with its maximum at 625 nm. The broad spectral feature is likely due to the relatively large imaginary permittivity and concomitantly large optical loss. Consequently, a substantial absorptance of 80–90 % is observed across the whole visible spectrum, making the patterned film twice as absorptive compared to unpatterned films. This enhancement indicates efficient light trapping and an increased interaction between incident light and the nanostructured film.

**Figure 3:**
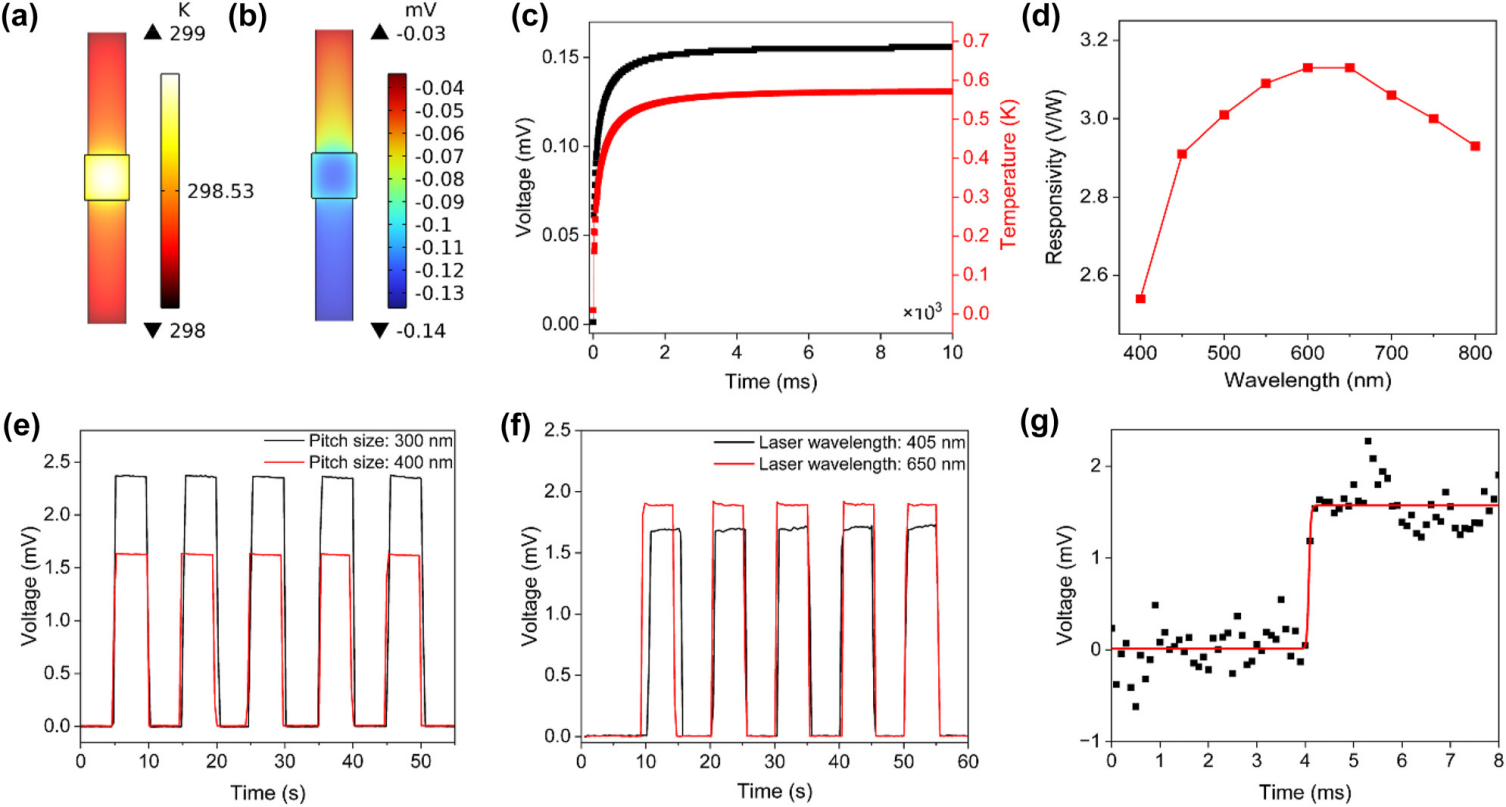
Thermoelectric simulations and photoresponse measurements. (a) A nonuniform temperature distribution is generated in the device due to photothermal heating. The central region gets hotter due to the enhanced absorptance of the nanostructure absorber. (b) The temperature gradient produces a thermoelectric potential along the device due to its *n*-type/*p*-type thermocouple configuration. (c) Simulated temperature and voltage response due to photothermal heating. The response time is on the order of 200 µs. (d) Simulated spectral responsivity. The responsivity is wavelength dependent due to the wavelength dependent optical absorptance. (e) Photoresponse measurement as an illuminating laser beam at a wavelength of 650 nm is turned on and off, comparing 300 and 400 nm pitch devices. (f) Photoresponse comparing laser wavelengths of 405 and 650 nm for the device with 300 nm pitch size. (g) High-resolution transient measurement, from which a rise time of 160 µs could be extracted.

In addition to optical absorption, a thermoelectric photodetector depends on the heat transfer within the device; therefore, multiphysics finite element simulations were performed to aid the thermal design. Experimental absorptance values and illumination intensities were used as inputs for the heat sources in the thermoelectric model, together with measured Seebeck coefficients of 164 and −85 μV/K for Sb_2_Te_3_ and Bi_2_Te_3_. Figure 3(a)–(c) shows thermoelectric simulations of the nanostructured thermoelectric device under uniform illumination. The hottest part is in the middle, resulting in a voltage difference (Δ*V*) between the hot region and the cold electrodes at either end. Heat flows from the central region (where the nanostructures are located) into the Sb_2_Te_3_ and Bi_2_Te_3_ pads on the sides of the device, into the substrate and the surrounding medium (air), leading to cooling and resetting of the detector once the illumination is removed. Figure 3(d) presents the simulation results for the responsivity of the proposed thermoelectric photodetector across the visible spectrum. The results show that the device achieves its highest responsivity at a wavelength of 625  nm, indicating that the photodetector is maximally sensitive to light at this wavelength.

Figure 3(e) shows our fabricated photodetector featuring a nanostructured absorber in its central region. To demonstrate the photodetection capability, the device was illuminated with a laser beam focused onto the absorber region. The resulting photovoltage due to the laser being turned on and off is shown in Figure 3(e), and it is apparent that the device is photosensitive. To investigate the influence of nanostructure pitch and, therefore, absorptance on device performance, we compared the photovoltage of two devices with pitches of 300 nm and 400 nm. The results demonstrate a higher voltage for the device with a 300 nm pitch. This observation is consistent with the finding that absorptance is enhanced with decreasing pitch (Figure S2(c)). Further analysis was conducted by measuring the photoresponse of the 300  nm pitch device under different laser illuminations, comparing wavelengths 405 nm and 650 nm, as shown in Figure 3(f). The power output of both lasers was tuned to ∼1 mW, and the detector responsivities for each wavelength were calculated to be 1.8 V/W at 405 nm and 2.5 V/W at 650nm. These results suggest that while the device is more responsive to light at 650 nm, it still maintains considerable sensitivity at 405 nm, matching its broad absorptance spectrum. The higher responsivity at 650 nm is attributed to the higher absorption (∼90 %) of the Sb_2_Te_3_ and Bi_2_Te_3_ nanoposts in the 600–650 nm wavelength range, and this trend agrees with the spectral responsivity simulation in Figure 3(d). The slightly higher simulated responsivities could be attributed to variations in material properties from the device fabrication. The response time of the thermoelectric photodetector was measured in Figure 3(g) to be ∼160 µs, which agrees with the time constant of ∼220 µs that can be extracted from the thermal simulation in Figure 3(c). This response time is competitive, agreeing well with other published works in the field [5].

**Figure 4:**
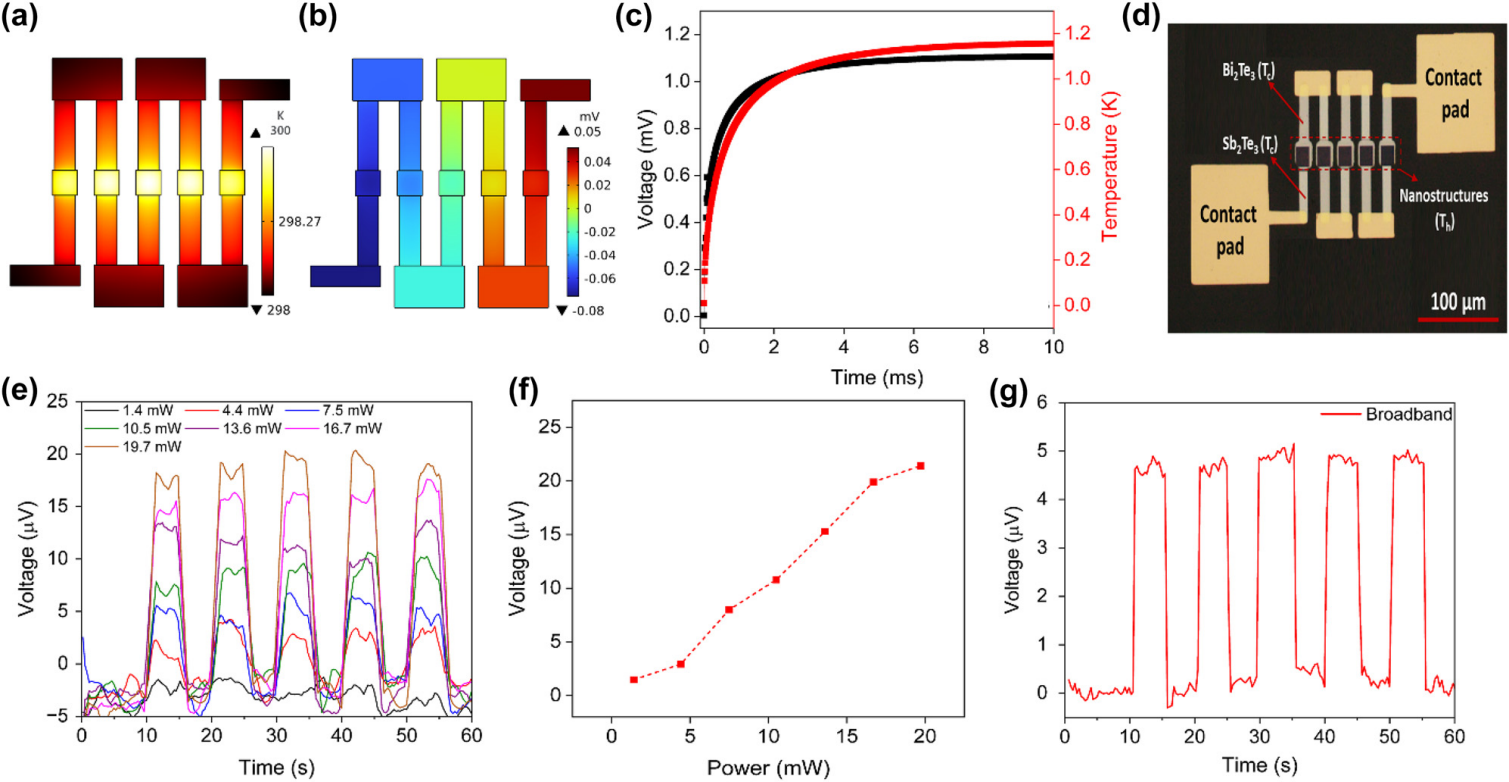
Photoresponse of a five-unit thermopile detector. (a) Simulated thermal distribution of a 5-unit thermopile detector. (b) Thermoelectric potential of the 5-unit device. (c) Simulated temperature and voltage difference of the 5-unit device. (d) Fabricated device with five serially connected thermocouples. The central array of dark squares is the nanostructure absorbers, from which alternating Sb_2_Te_3_ and Bi_2_Te_3_ legs are extended outward. The gold contact pads are used for electrical measurements. (e) Photoresponse due to unfocused laser illumination and different laser powers. (f) Power-dependent photoresponse. (g) Photoresponse due to broadband illumination from a halogen lamp with an intensity of ∼18,000 W/m^2^.

The photoresponse of the detector in Figure 3 can be further enhanced by connecting multiple units in series. The resulting device is commonly referred to as a thermopile. The simulation of multiple cascaded thermoelectric devices (Figure 4(a)–(c)) shows an increase in voltage difference proportional to the number of devices cascaded together (*N* × Δ*V*). The cascading of multiple devices results in a cumulative increase in voltage difference, indicating enhanced electrical output. Furthermore, the neighboring absorbers in the cascaded configuration facilitate heat transfer and redistribution across the devices. This redistribution of heat helps to mitigate losses by harvesting the excess heat from neighboring devices, thereby improving the overall efficiency of the thermoelectric system. The findings highlight the importance of device architecture and configuration in optimizing thermoelectric device performance. Cascading multiple thermoelectric elements, with all elements ideally arranged in the central region as shown in Figure 4(d), enables the collective harvesting of heat and enhances electrical output. This configuration is a favorable strategy for improving device efficiency. Efficient heat transfer and utilization across the cascaded devices contribute to minimizing heat losses and maximizing energy conversion efficiency.

The experimental results of our cascaded thermoelectric photodetector, composed of Sb_2_Te_3_ and Bi_2_Te_3_ nanoposts with a 300 nm pitch size, offer valuable insights into the device performance and sensitivity. The photoresponse of a 5-unit thermopile device was measured under uniform illumination using a collimated 650 nm laser beam with a diameter 2.4 mm, applied at varying power levels (Figure 4(e)). At an input power of 19.7 mW, the thermopile voltage was 21 µV, corresponding to a responsivity of 1.5 V/W when adjusted for light incident on total active area of the device. While this responsivity is 40 % lower compared to the one measured using focused light – likely due to additional photothermal heating of the unpatterned legs – the result shows that the device also operates well under full area illumination. To evaluate the noise performance, we assume the noise floor to be electrical Johnson noise, which is typically what thermal detectors are limited by. The Johnson-limited specific detectivity *D* ^*^ can be calculated as $D^{*}=\frac{R\sqrt{A}}{\sqrt{4kTR}}$ , where *k* is the Boltzmann constant, *T* is the operating temperature of 300 K, and *A* is the area of the photosensitive region, which was taken as 120 µm^2^ from the rectangular region encompassing the five absorbers. The electrical resistance, *R*, was determined to be 25 kΩ, as measured from the IV curves under dark conditions, as shown in Figure S4(a). The detectivity for full-area illumination was found to be $3.2\times 10^{6}\;\text{cm H}{\text{z}}^{1/2}\;{\text{W}}^{-1}$ for a wavelength of 650 nm. Despite the lack of thermal isolation from the substrate, this detectivity is formidable compared to devices made on thin membrane [6]. A comparison of different thermoelectric photodetectors is listed in Table S1. The thermoelectric nanopost array presented in this work provides a key advantage over previous designs by directly fabricating the nanoresonators from the thermoelectric materials (Bi_2_Te_3_/Sb_2_Te_3_), enabling efficient direct absorption of incident light. This design eliminates the need for intermediate layers or additional absorption-enhancing components, which can introduce thermal resistance and slow down heat transfer. This approach allows for immediate photon-to-heat conversion within the active material, minimizing energy loss and reducing the thermal diffusion time, leading to a faster photothermal response.

The responsivity of the photodetector was observed to be 1.5 V/W for all the measured input powers, as indicated by the linear scaling of the photovoltage in Figure 4(f). This direct proportionality between output voltage and input light intensity supports the photodetector sensitivity and potential for scalable signal output based on light intensity. The photoresponses of single and five-unit thermopile detectors under uniform illumination using an unfocused 650 nm laser were measured (Figure S4(b)). The results show larger voltages for the five-unit device compared to the single-unit device. We also measured the photoresponse of a five-unit thermopile detector under laser illumination focused on individual subdevices. As shown in Figure S4(c) and (d), the voltage difference is larger when the middle device is illuminated. In Figure 4(g), we investigated the photoresponse of the cascaded photodetector when exposed to a broadband light source covering the entire visible spectrum with an intensity of ∼18,000 W/m^2^. The total incident power on the device was adjusted according to the power spectral density of the halogen lamp (see Supplementary Information), and the responsivity was calculated to be 1.5 V/W, which is comparable with the response to coherent light. The result indicates that the device is responsive to visible light across a broad range of wavelengths, confirming its applicability in multiwavelength detection.

## 4 Conclusions

This work offers a new look at the traditional thermoelectric materials Sb_2_Te_3_ and Bi_2_Te_3_ and their recently discovered ability to support interband plasmonic resonances – in the context of photodetection. By fabricating nanoresonators directly from the thermoelectric materials, we show that plasmonic field localization enhances the optical absorptance in a thermocouple to 90 percent across the visible spectrum. The direct photo-thermoelectric conversion within the materials eliminates the need for external nanoengineered materials or black-body coatings to collect photons, resulting in reduced device complexity and bulk. Importantly, the reduced heat capacitance improves the speed of the device, which is generally a limitation of thermal-type photodetectors.

In contrast to metallic nanostructures that typically support plasmonic resonances with narrow spectral features, the interband resonances observed here are broad with high absorptance across the visible spectrum. The broad resonances are owed to the large absorptive losses of the thermoelectric materials and could be advantageous for thermoelectric photodetectors as they are commonly used for broadband applications.

As demonstrated here, nanophotonic engineering is a powerful tool to improve the functionality and performance of optoelectronic devices. By applying mature nanofabrication techniques like electron beam lithography to unconventional materials like thermoelectrics, new advancements can be made to align optical properties with application-specific requirements. Against this backdrop, we foresee that thermoelectric nanoresonators, like the one presented here, offer a promising path forward for various optoelectronic technologies, such as imaging, sensing, solar energy harvesting, and integrated spectrometers.

## Supplementary Material

Supplementary Material Details

## References

[1] Qiu Q., Huang Z. (2021). Photodetectors of 2D materials from ultraviolet to terahertz waves. *Adv. Mater.*.

[2] Wang R., He Z., Wang J.-L., Liu J.-Y., Liu J.-W., Yu S.-H. (2022). Manipulating nanowire structures for an enhanced broad-band flexible photothermoelectric photodetector. *Nano Lett.*.

[3] Wang J., Xie Z., Yeow J. T. (2020). Two-dimensional materials applied for room-temperature thermoelectric photodetectors. *Mater. Res. Express*.

[4] Lu X., Jiang P., Bao X. (2019). Phonon-enhanced photothermoelectric effect in SrTiO3 ultra-broadband photodetector. *Nat. Commun.*.

[5] Mauser K. W. (2017). Resonant thermoelectric nanophotonics. *Nat. Nanotechnol.*.

[6] Sharma N., Bar-David J., Mazurski N., Levy U. (2020). Metasurfaces for enhancing light absorption in thermoelectric photodetectors. *ACS Photonics*.

[7] Dai M., Zhang X., Wang Q. J. (2024). 2D materials for photothermoelectric detectors: mechanisms, materials, and devices. *Adv. Funct. Mater.*.

[8] Chen J., Ying X. (2024). High-performance, ultra-broadband Sb2Te3 photodetector assisted by multimechanism. *AIP Adv.*.

[9] Hu Y., Wang Y., Sang T., Yang G. (2023). Mid-infrared circular-polarization-sensitive photodetector based on a chiral metasurface with a photothermoelectric effect. *Appl. Opt.*.

[10] Wredh S. (2024). Sb2Te3–Bi2Te3 direct photo–thermoelectric mid‐infrared detection. *Adv. Opt. Mater.*.

[11] Zhang X., Zhao L.-D. (2015). Thermoelectric materials: energy conversion between heat and electricity. *J. Mater.*.

[12] Yuan Z., Wu P. C., Chen Y. C. (2022). Optical resonator enhanced photovoltaics and photocatalysis: fundamental and recent progress. *Laser Photonics Rev.*.

[13] Yin J. (2017). Plasmonics of topological insulators at optical frequencies. *NPG Asia Mater.*.

[14] Dong Z. (2019). Ultraviolet interband plasmonics with Si nanostructures. *Nano Lett.*.

[15] Lin K.-T., Lin H., Jia B. (2020). Plasmonic nanostructures in photodetection, energy conversion and beyond. *Nanophotonics*.

[16] Yang J. K., Berggren K. K. (2007). Using high-contrast salty development of hydrogen silsesquioxane for sub-10‐nm half-pitch lithography. *J. Vac. Sci. Technol., B: Microelectron. Nanometer Struct. Process., Meas., Phenom.*.

[17] Piccinotti D., Gholipour B., Yao J., MacDonald K. F., Hayden B. E., Zheludev N. I. (2019). Stoichiometric engineering of chalcogenide semiconductor alloys for nanophotonic applications. *Adv. Mater.*.

[18] Thiyagarajah N. (2012). Effect of inter-bit material on the performance of directly deposited bit patterned media. *Appl. Phys. Lett.*.

[19] Ghosh S. K., Pal T. (2007). Interparticle coupling effect on the surface plasmon resonance of gold nanoparticles: from theory to applications. *Chem. Rev.*.

[20] Wang W., Qi L. (2019). Light management with patterned micro‐and nanostructure arrays for photocatalysis, photovoltaics, and optoelectronic and optical devices. *Adv. Funct. Mater.*.

