# Supplementary material for: Interband plasmonic nanoresonators for enhanced thermoelectric photodetection
Source: Nanophotonics. 2025 Mar 28;14(9):1365–73. doi: 10.1515/nanoph-2024-0752 (PMC12038571; doi:10.1515/nanoph-2024-0752)
Supplement: Supplementary file 1 — Supplementary Material Details [file j_nanoph-2024-0752_suppl_001.pdf]

Supplementary Information

Interband Plasmonic Nanoresonators for Enhanced Thermoelectric Photodetection

Golnoush Zamiri[1†], Simon Wredh[1†], Md Abdur Rahman[1], Nur Qalishah Adanan[1], Cam Nhung Vu[1], Wang Hong Tao[1], Deepshikha Arora[1], Wakana Kubo[2], Zhaogang Dong[3], Robert E. Simpson[4], Joel W.K Yang[1,5]*

1 Singapore University of Technology and Design, 8 Somapah Road, Singapore 487372

2 Tokyo University of Agriculture and Technology, Faculty of Engineering, Koganei, Tokyo, Japan

3 Institute of Materials Research and Engineering (IMRE), Agency for Science, Technology and Research (A*STAR), 2 Fusionopolis Way, Innovis #08-03, Singapore 138634, Republic of Singapore

4 University of Birmingham, Edgbaston, B15 2TT, UK

5 Singapore-HUJ Alliance for Research and Enterprise (SHARE), The Smart Grippers for Soft Robotics (SGSR) Programme, Campus for Research Excellence and Technological Enterprise (CREATE), Singapore 138602, Singapore

† G. Zamiri and S. Wredh contributed equally to this work.
* Corresponding author: joel_yang@sutd.edu.sg

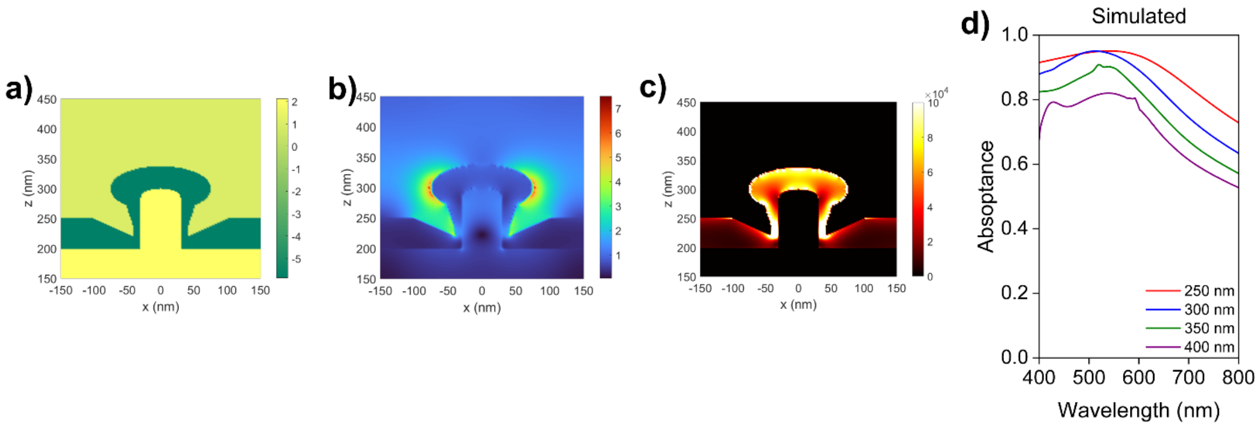

Fig. S1: (a, b, c) Real permittivity, absorbed power density and electric field intensity of Sb_2Te_3 nanostructures at λ=536 nm. (d) Simulated absorptance spectra for Sb_2Te_3 nanostructure arrays.

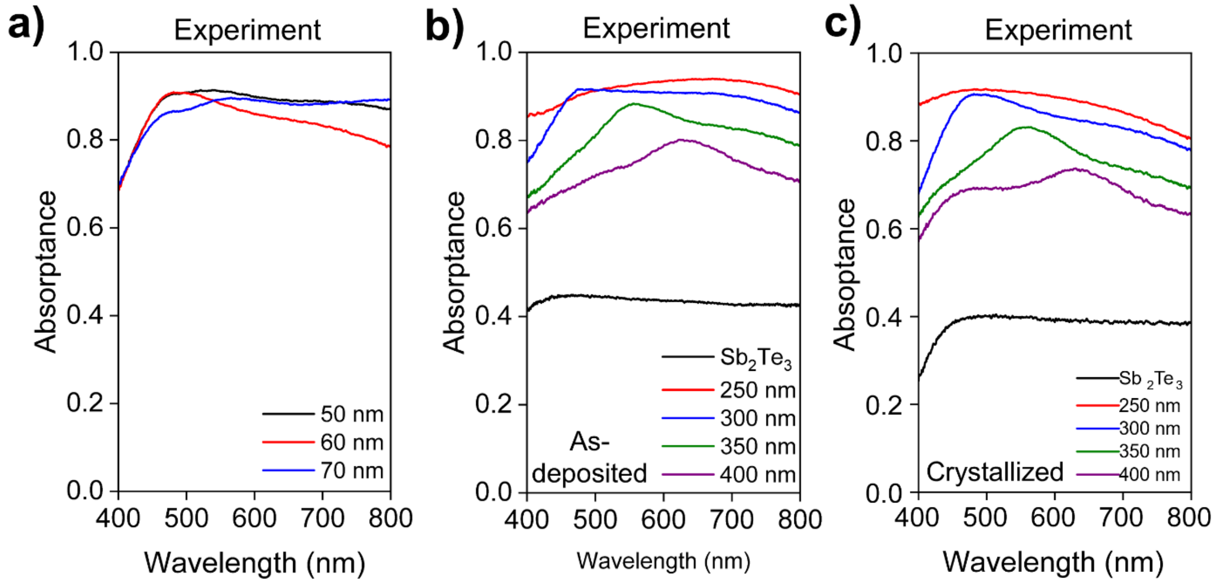

Fig. S2: (a) Optical absorptance spectra of fabricated nanoposts arrays of the 300 nm pitch with varying sizes. (b) Optical absorptance spectra of nanoposts arrays made of as-deposited Sb_2Te_3 with varying pitch. (c) Optical absorptance of nanoposts arrays made of crystallized Sb_2Te_3 with varying pitch.

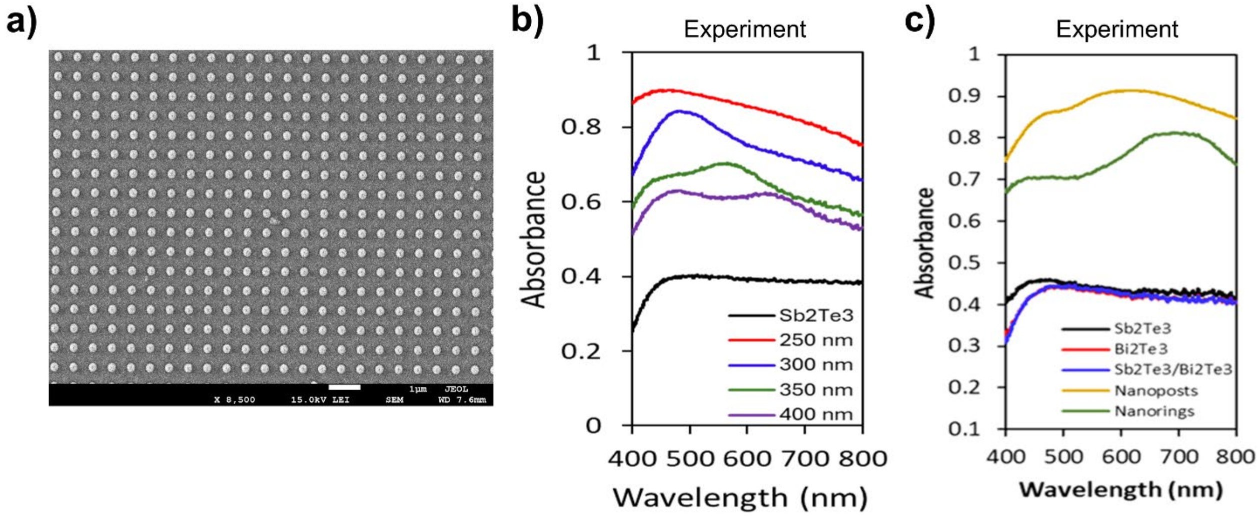

Fig. S3: (a) Scanning electron micrograph of fabricated Sb_2Te_3 nanoholes. (b) Optical absorptance spectra of nanoholes arrays with varying pitch. (c) absorptance spectra for Sb_2Te_3, Bi_2Te_3, Sb_2Te_3/Bi_2Te_3 plain films and Sb_2Te_3/Bi_2Te_3 nanostructure arrays.

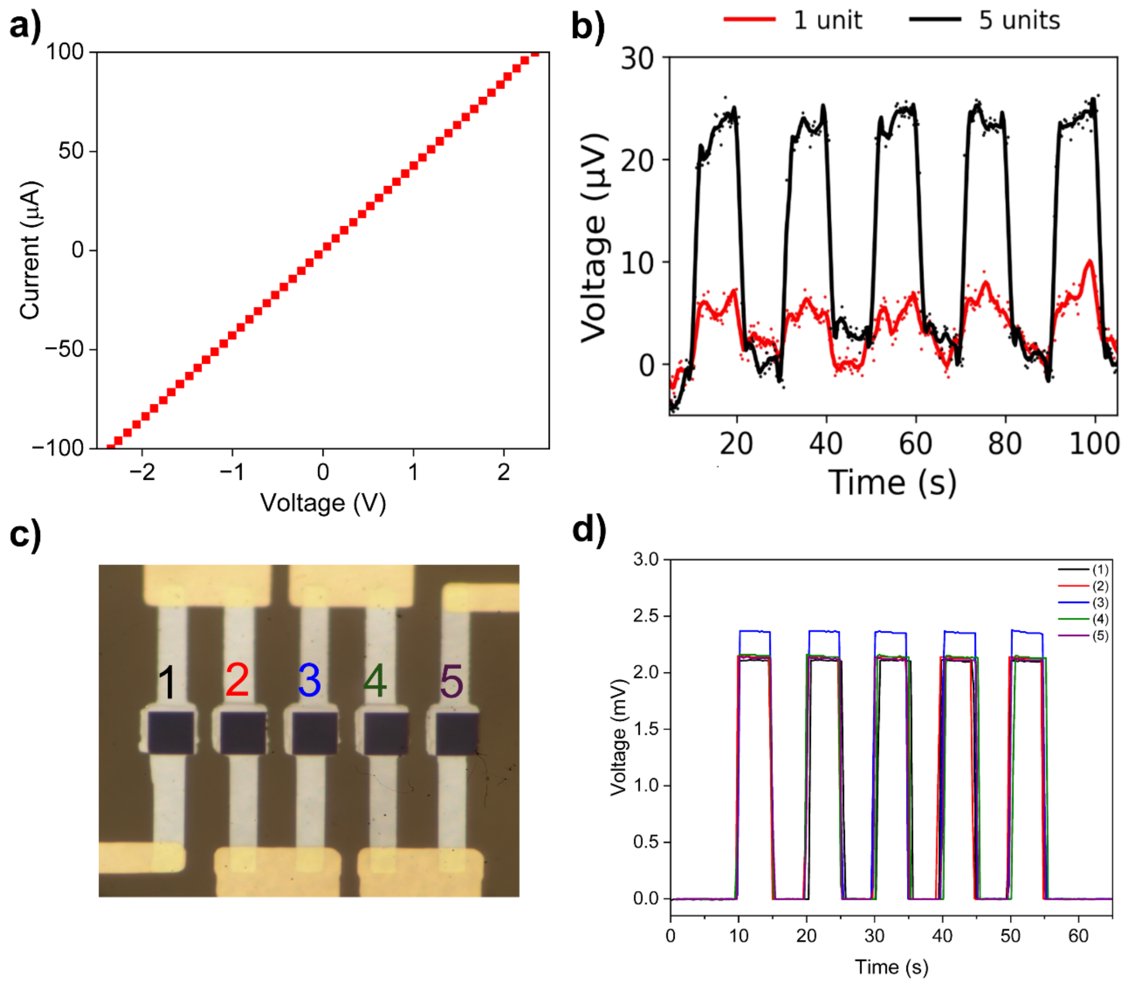

Fig. S4: a) IV curves under dark conditions. b) Photoresponse of single and five-unit thermopile detector under uniform illumination using an unfocused 650 nm laser. c) Fabricated device with five serially connected thermocouples for photoresponse measurements. d) Photoresponse of a five-unit thermopile detector under laser illumination focused on individual sub-devices, named as 1 to 5 simultaneously.

Thermal diffusion length

While it is observed in the main manuscript that the absorption of heat is localized in hotspots at the base of the nanostructures, it is reasonable to believe that the temperature distribution in the nanostructures will rapidly thermalize due to the short length scales. The thermal diffusion time can be estimated using the Einstein-Smoluchowski equation $\tau = \frac{L^2}{D}$, where L is the spatial length and D is the thermal diffusivity. The thermal diffusivity is given by $D = \frac{\kappa}{\rho c_p}$, where κ is the thermal conductivity, ρ is the density and c_p is the specific heat capacity. Typical values for Sb_2Te_3 and Bi_2Te_3 are $\kappa \approx 1$ W/(m*K) and $c_p \approx 200$ J/(kg*K), with densities $\rho_{Sb2Te3} = 6500$ kg/m^3 and $\rho_{Bi2Te3} = 7700$ kg/m^3. From these values we can get a lower estimate of the thermal diffusivity of the double-layered nanostructures as $D = \frac{1}{200 \cdot 7700} \approx 6.5 \cdot 10^{-7}$ m^2/s. Assuming that the heat must diffuse a distance of $L = 100$ nm to cover the whole nanostructure, the diffusion time can be estimated to be $\tau = \frac{(100 \cdot 10^{-9})^2}{6.5 \cdot 10^{-7}} \approx 15 \cdot 10^{-9}$ seconds. Hence, it is reasonable to

assume that the temperature distribution in the nanostructures will become homogenous in 10s of nanoseconds.

Halogen lamp responsivity

A microscope halogen lamp (Nikon Halogen 12V50W LV-LH50PC) focused using a 50x 0.4 NA lens was used to measure the photoresponse of the devices under broadband illumination. The beam diameter was taken as the FOV diameter seen through the microscope eyepiece, and it was measured to be 0.42 mm. The broadband illumination power was measured using a power meter (Ophir PD300) and adjusted according to the spectral power density of the halogen source to be 2.5 mW. The intensity was then calculated as $I = \frac{P}{\pi r^2} = 18000$ W/m^2. With a measured photovoltage of 45 µW and 5 thermocouple units of 18x18 µm areas, the resulting responsivity was calculated as $\mathcal{R} = \frac{45 \cdot 10^{-6}}{18000 \cdot 5 \cdot (18 \cdot 10^{-6})^2} \approx 1.5$ V/W.

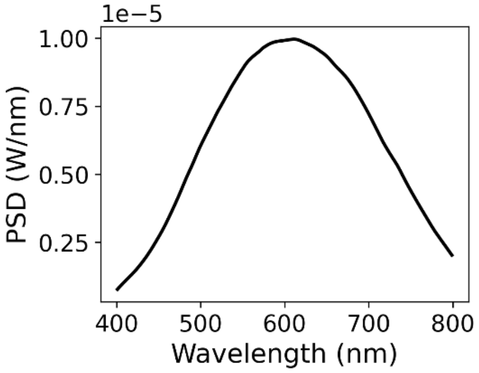

Fig. S5: Spectral distribution of halogen lamp.

Thermal simulation time constant

A time constant of $\tau = 220$ µs was extracted from the thermal simulations by fitting the temperature difference data to the function $\Delta T(t) = \Delta T_0 (1 - e^{-\frac{t}{\tau}})$.

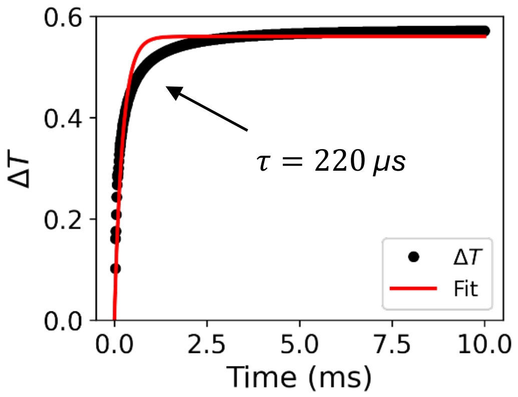

Fig. S6: Thermal time response of the single-unit photodetector.

Table S1: Comparison of thermoelectric photodetectors with resonant absorption

Material	Resonant absorber type	Responsivity (V/W)	Detectivity $cmHz^{1/2}\,W^{-1}$	Response time	Spectral range (nm)	Ref
Bi_2Te_3/Sb_2Te_3	Thermoelectric nanopost array	1.5	3.2×10^6	160 µs	400-800	This work
Bi_2Te_3/Sb_2Te_3	Thermoelectric grating	38	-	360 µs	400-800	[1]
Bi_2Te_3/Sb_2Te_3	Thermoelectric Fabry-Perot	10.2	4.6×10^6	460 µs	2000-10000	[2]
Au-Bi	Metallic grating	0.26	1.3×10^8	3.9 s	400-800	[3]
Au–graphene	Dielectric hole array	8	-	23 ms	6000-14000	[4]
Bi_2Te_3/Sb_2Te_3	Suspended dielectric Fabry-Perot	7000	4.4×10^9	58 ms	8000-12000	[5]

References

[1] K. W. Mauser *et al.*, "Resonant thermoelectric nanophotonics," *Nature nanotechnology,* vol. 12, no. 8, pp. 770-775, 2017.

[2] S. Wredh *et al.*, "Sb2Te3–Bi2Te3 Direct Photo–Thermoelectric Mid‐Infrared Detection," *Advanced Optical Materials,* p. 2401450, 2024.

[3] H. Monshat, L. Liu, and M. Lu, "A narrowband photo‐thermoelectric detector using photonic crystal," *Advanced Optical Materials,* vol. 7, no. 3, p. 1801248, 2019.

[4] A. L. Hsu *et al.*, "Graphene-based thermopile for thermal imaging applications," *Nano Lett.,* vol. 15, no. 11, pp. 7211-7216, 2015.

[5] A. S. Gawarikar, R. P. Shea, and J. J. Talghader, "High detectivity uncooled thermal detectors with resonant cavity coupled absorption in the long-wave infrared," *IEEE Trans. Electron Devices,* vol. 60, no. 8, pp. 2586-2591, 2013.
